# Screen-Printing vs Additive Manufacturing Approaches: Recent Aspects and Trends Involving the Fabrication of Electrochemical Sensors

**DOI:** 10.1021/acs.analchem.4c05786

**Published:** 2025-01-16

**Authors:** Luiz O. Orzari, Cristiane Kalinke, Habdias A. Silva-Neto, Danielly S. Rocha, Jéssica R. Camargo, Wendell K.T. Coltro, Bruno C. Janegitz

**Affiliations:** † Department of Nature Sciences, Mathematics and Education, Federal University of São Carlos, 13600-970 Araras, São Paulo, Brazil; ‡ Department of Physics, Chemistry and Mathematics, Federal University of São Carlos, 18052-780 Sorocaba, São Paulo, Brazil; § Institute of Chemistry, University of Campinas, 13083-859 Campinas, São Paulo, Brazil; ∥ Department of Chemistry, Federal University of Parana, 81531-980 Curitiba, Paraná, Brazil; ⊥ Institute of Chemistry, Federal University of Goiás, 74690-900 Goiânia, Goiás, Brazil; # Department of Chemistry, Federal University of Santa Catarina, 88040-900 Florianópolis, Santa Catarina, Brazil; ∇ National Institute of Bioanalytical Science and Technology, 13084-971 Campinas, São Paulo, Brazil

## Abstract

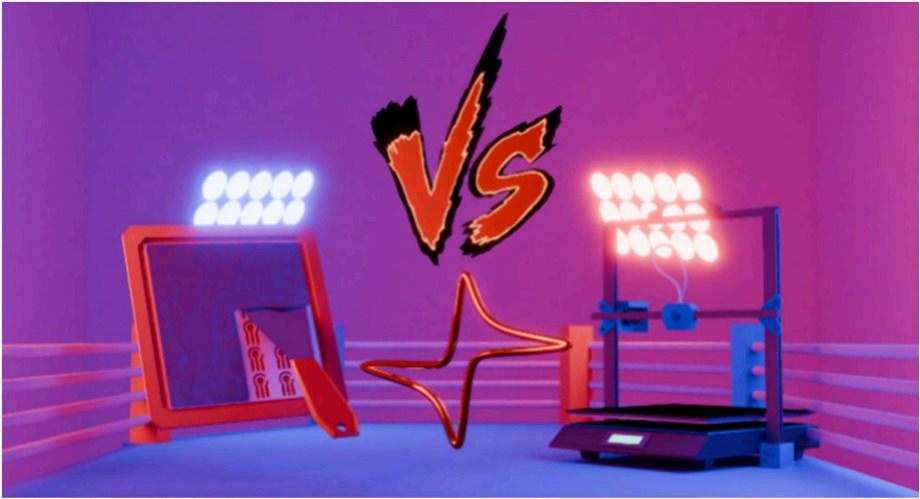

A few decades ago, the technological boom revolutionized access to information, ushering in a new era of research possibilities. Electrochemical devices have recently emerged as a key scientific advancement utilizing electrochemistry principles to detect various chemical species. These versatile electrodes find applications in diverse fields, such as healthcare diagnostics and environmental monitoring. Modern designs have given rise to innovative manufacturing protocols, including screen and additive printing methods, for creating sophisticated 2D and 3D electrochemical devices. This perspective provides a comprehensive overview of the screen-printing and additive-printing protocols for constructing electrochemical devices. It is also informed that screen-printed sensors offer cost-effectiveness and ease of fabrication, although they may pose challenges due to the use of toxic volatile inks and limited design flexibility. On the other hand, additive manufacturing, especially the fused filament fabrication (or fused deposition modeling) strategies, allows for intricate three-dimensional sensor designs and rapid prototyping of customized equipment. However, the post-treatment processes and material selection can affect production costs. Despite their unique advantages and limitations, both printing techniques show promise for various applications, driving innovation in the field toward more advanced sensor designs. Finally, these advancements pave the way for improved sensor performance and expand possibilities for academic, environmental, and industrial applications. The future is full of exciting opportunities for state-of-the-art sensor technologies that will further improve our ability to detect and determine various substances in a wide range of environments as researchers continue to explore the many possibilities of electrochemical devices.

## Introduction

The electrochemical sensors are instruments that detect and determine chemical species by monitoring the progress of electrochemical processes. Such technology has an impact on several industries.^1,2^ For example, they can be used by healthcare facilities to measure the blood sugar levels of diabetic patients.^1,3^ Also, they can be employed to identify potential contaminants in environmental samples, including water or industrial waste.^1,2,4,5^ Thus, this is a fast-advancing field where researchers are investigating various sensor fabrication techniques to improve a specific device’s accuracy, efficiency, and versatility. Of these techniques, two have gained traction in recent literature: screen-printing and additive manufacturing.^6−10^ Both techniques have advantages and disadvantages specific to the problem of producing electrochemical sensors. It is essential to comprehend these techniques and their consequences to choose the right technology for a given application. The printing techniques explored in this report have a great impact on modern academy, environment, and industry, and a brief comment on the topic can be read in the Supporting Information.

Screen-printing is a very simple technique: conductive ink is applied over a substrate by spreading it through a mesh screen. The resulting device has each electrode shape determined by the geometry of the screen. As such, this process is desirable for repeatable, highly cost-efficient, and inexpensive mass production of electrochemical devices with a relatively uniform quality. These sensors are commonly flexible and, due to their simplicity, they may be easily integrated into other devices.^11,12^ Therefore, they are extensively employed in industries, such as environmental analysis^13−15^ and wearable health monitor devices^16,17^ and other portable gadgets.^18,19^ Single-use applications are made easier by the use of disposable materials, without decreasing efficiency or requiring extensive analysis time.^20,21^ A short analysis discussing how screen-printing fares in comparison with other commonly employed two-dimensional electrode fabrications can be found in the Supporting Information.

On the other hand, a relatively new approach to sensor fabrication is additive manufacturing.^22−24^ By employing digital models, this technique is based on building an electrode layer by layer. Complex geometries and specifically shaped sensors can be easily prototyped, which cannot be achieved by conventional fabrication methods. Through the use of composite inks and polymers, new filament compositions can be easily produced, leading to further novelty and electrodes with distinct qualities, such as flexibility^25−27^ and hydrophobicity.^28,29^ Being able to change the shapes and architectures of electrochemical sensors has several advantages mainly for the miniaturization of devices. For instance, electrodes may be tailored for specific applications, such as body implants^30^ or devices with detailed surface areas, for higher sensitivity.^10^ Moreover, the mixture of many materials in the printing procedure might lead to the development of multifunctional devices, combining electrical and structural contributions to the system sensing capability.

Therefore, the goal of this review is to critically examine and contrast these two approaches for manufacturing electrochemical sensors. Through a thorough analysis of their different materials, characteristics, and uses, this review intends to provide an in-depth assessment that will guide further studies and advancements in the field. This paper sheds light on how each method improves sensor technology and helps determine which fabricating strategy is best for a given set of sensing requirements.

## Screen-Printed Electrochemical Sensors

Screen-printing is a technique where ink or other materials are forced through a fine-mesh screen onto a surface to produce a specific pattern or design.^8,31−33^ Screen-printing has been used for decades in various industries, such as textile printing,^34^ electronics,^35^ and packaging.^36^ This level of maturity ensures a reliable and consistent manufacturing process. In this context, screen-printing is one of the most promising approaches for the simple, fast and cost-effective production of sensors and biosensors.^37^ Silva et al.,^38^ Suresh et al.,^11^ and Paimard et al.^39^ showed that one of the main advantages of this technique for producing screen-printed electrodes (SPEs) is its cost efficiency. The equipment and materials required for the process are relatively inexpensive, and the technique enables the rapid and large-scale production of the electrodes. This makes it ideal for large-scale production and promotes a reduction of the overall cost per unit.^40^ The advantages mentioned above, such as the associated low cost and ease of processing, have drawn attention to the creation of new SPE systems, which can also be modified depending on the intended use. Screen-printing offers considerable flexibility in modifying both the surface and the ink used.^39,41,42^ The ink formulations can be customized to achieve specific properties required for different sensor applications.^43^ In addition, surface modifications can improve adhesion and sensor performance, increasing the versatility of sensor design and functionality.^44^

SPE sensors have been using the benefit of the compatibility of the screen-printing process with a wide range of materials. This technique can be used on various substrates, including paper,^45^ textiles,^46^ glass,^47^ ceramics,^48^ and different polymers.^49^ This compatibility enables the development of sensors for a wide range of applications in many industries. Not only must the substrate be chemically and physically resistant to the sample investigated but also the interaction between substrate and ink can lead to different structural configurations of the conductive particle, leading to different responses.^50,51^ Also, the choice of substrate is very important when intending a more eco-friendly approach for a given device.^51^

On the other hand, there are some disadvantages in the fabrication of SPE systems that need to be addressed, if possible, to boost and promote their real-time usage in situational needs. For example, the screen-printing technique has limitations in terms of resolution and feature size.^52^ SPEs may not achieve the level of detail and precision required for some applications and miniaturized systems, as the process generally cannot produce structures smaller than a few tens of micrometers.^53^ Also, screen-printing does not currently have any easy means of changing the printing resolution. Moreover, ensuring reproducibility across different batches can be challenging. Intrinsically, the method employs manual dispersion, causing issues: small variations in the printing process can lead to inconsistencies in sensor performance and affect their reliability. This is especially true when developing impedimetric experiments, as small variations on the surface can cause great impact on double-layer capacitance.^54^ Variability between batches of SPE sensors can be caused by factors such as differences in ink composition, substrate properties, and printing conditions, leading to inconsistencies in sensor performance and reproducibility.^54,55^ There are ways of increasing reproducibility, however: by using specialized machinery, it is possible to standardize the mixture of the ink formulation, as well as the distribution of the ink over the desired substrate.^55,56^ It is important to highlight that this includes manual-printing-based printers in production lines, as they are a cost-effective solution with high reproducibility. In addition, when high performance is key, different modification agents can be used, such as molecularly imprinted polymers or metallic particles, helping to level the response obtained and diminishing variations. The downside to these alternatives is that they incur a cost in resources, sometimes drastically increasing the cost per device or time to produce a batch.^54−59^

Although there are various substrate options available, the choice of substrate is critical to the performance and quality of the SPE devices. Not all substrates are suitable for screen-printing, and some of them require extensive pretreatment to achieve the desired sensor properties.^60^ Usually, a desirable substrate has the following characteristics: it is chemically inert, has controllable surface roughness, and must adhere to the ink. Also, if flexibility is a desired property, then the substrate must be flexible as well. In addition, the integration of multiple layers of ink can be complex and requires precise alignment and control to avoid issues such as misalignment or bleeding of ink between layers.^61^ The performance of the conductive ink can be significantly affected by fluctuations in the humidity and temperature. These changes can affect the viscosity, drying time, adhesion properties, and ultimately the conductivity of the ink.^35,62^ Therefore, it is critical to consider the environmental conditions during the printing and curing process to ensure consistent and reliable performance of conductive ink applications.^62^

**Figure 1:**
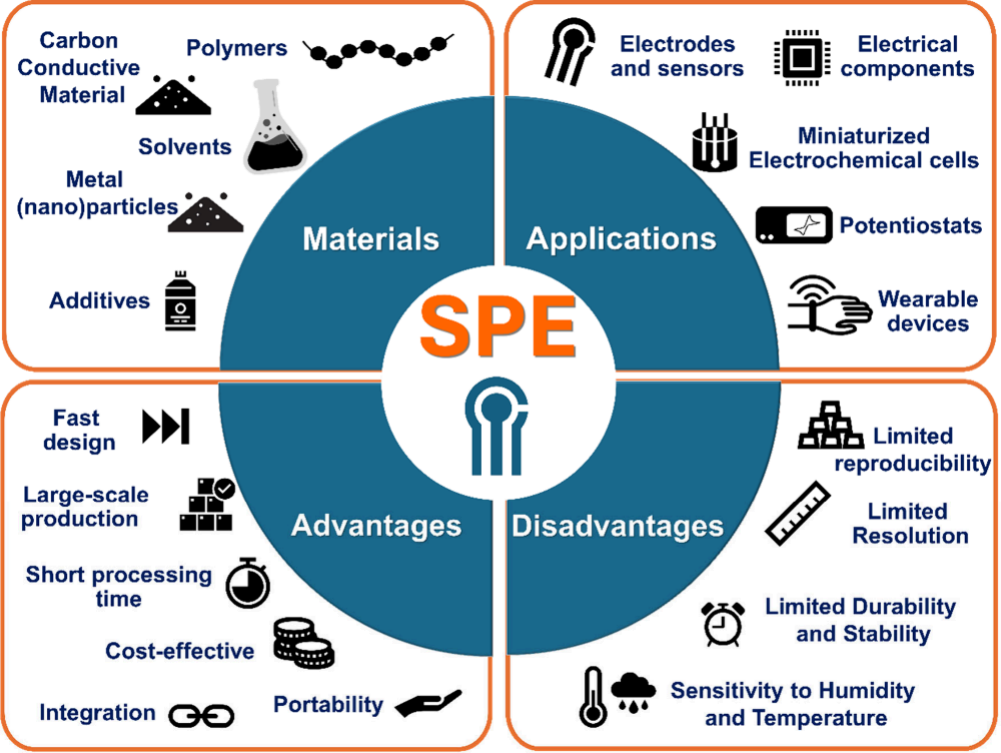
Summary of SPE materials, applications, and main advantages and disadvantages.

In this context, it can be stated that SPE devices are gaining popularity in various applications due to their numerous advantages and some inherent limitations, as can be seen in Figure 1.

The flexibility of materials that may be used to create electrochemical cells is a notable feature of screen- and stencil-printed electrodes.^63^ Regarding substrates, the literature frequently reports the use of ceramic materials, different kinds of paper, and polymeric films.^63^ In terms of paper platforms, chromatographic,^64^ filter,^65^ photo,^66^ stone,^67^ vegetal,^68^ waterproof,^69^ and office papers^70^ are usual examples. In turn, thermoplastic materials widely used for constructing electrochemical sensors are polyester,^71^ polyethylene terephthalate (PET),^72^ polyimide film,^73^ and transparency films.^74^

Another notable aspect in the realm of microfabrication dedicated to developing electrochemical devices is the vast variety of conductive inks available for use, which encompasses both commercialized and lab-made options, especially those based on carbon compounds or metallic nanoparticles.^75^ Graphite, carbon black, graphene, and carbon nanotubes have been employed due to their low cost, conductive properties, large potential window, and compatibility with (bio)chemical alterations.^76−79^ Carbon nanomaterial-based formulations frequently give conductive inks with faster electrode transfer rates than graphite alone because of their increased surface area and electrical conductivity. However, it is vital to note that the binder in the ink formulation might interfere with electron transport. To increase analytical performance, it may be essential to execute treatments on the working electrode, such as electrochemical activation, laser and plasma treatments, or mechanical polishing.^80−84^

Silver, copper, and gold are featured in conductive inks made by using metallic components (including nanoparticles) because of their exceptional qualities, such as high conductivity and low electrical resistance. However, compared to carbon-based inks, the cost is higher, particularly for silver and gold.^85−87^ In terms of analytical performance, they are more prone to oxidation, which can result in a drop in the electron transfer rate, affecting analytical performance.^11^ Thus, the composition of the ink chosen to build electrochemical sensors has a considerable impact on the performance of the device.^88^

Numerous configurations and geometries of 2D sensors are available commercially and in laboratory research contexts. The most common arrangement consists of three electrode systems composed by working, reference, and auxiliary electrodes.^76,89,90^ However, designs with more than one working electrode, multiple shapes (circular, linear, square, etc.), varied sizes, and other compositions and/or surface changes are also available. In terms of applicability, small samples (μL) can be used or the device can be submerged in an electrolyte solution. Furthermore, flexible and biocompatible technologies might be investigated as wearable sensors.^91^ Moreover, screen-printed electrodes are compatible with flow-batch analysis systems and microfluidic systems with the appropriate adjustments.^92^ All the aforementioned particularities inherent to screen-/stencil-printed electrodes allow these electrochemical sensors to be successfully applied in various areas, such as environmental,^73^ pharmaceutical,^93^ and clinical.^70,94^

**Figure 2:**
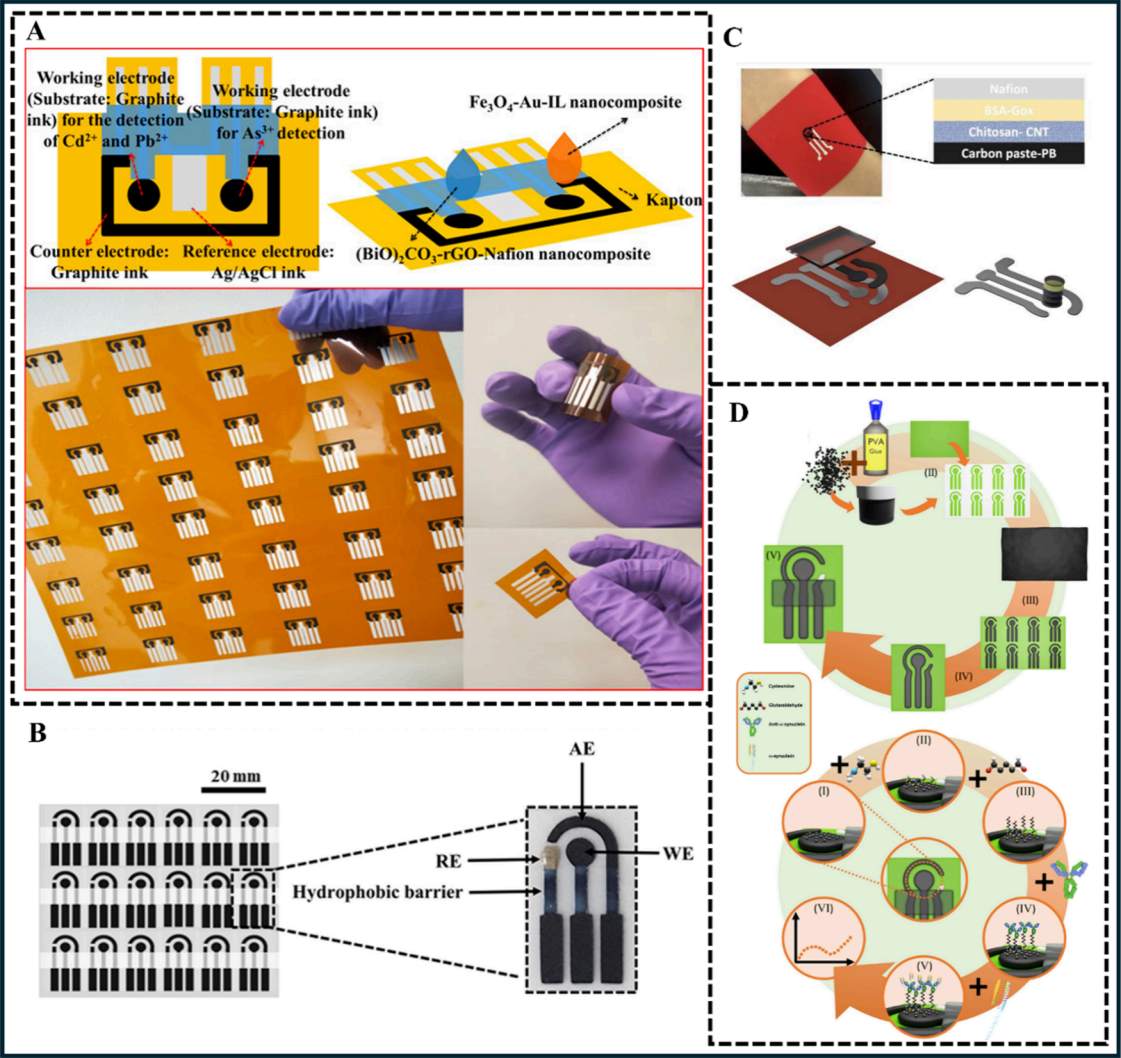
(A) Schematic representation of the screen-printed carbon electrode and real images of electrochemical sensors under different perspectives. Adapted with permission from ref (73),Copyright 2022 Frontiers Media SA. (B) Schematic representation showing the geometry of a disposable electrochemical paper-based analytical device based on graphite and polyester resin and a real picture of the sensor. Adapted with permission from ref (93). Copyright 2023 Springer Nature. (C) Steps involved on modification of electrochemical sensor and architecture of textile-based sensor for glucose. Used with permission from ref (17), Copyright 2023 MDPI. (D) Scheme of screen-printed electrodes of carbon black and poly(vinyl alcohol) and step-by-step of the WE (bio)chemical modification with (I) Pd nanoparticles, (II) cysteine, (III) glutaraldehyde, (IV) anti-α-synuclein, and (V) α-synuclein and electrochemical analysis by electrochemical impedance spectroscopy. Used with permission from ref (94), Copyright 2023 Springer Nature.

Zhao et al.^73^ demonstrated a screen-printed carbon electrode with the configuration of two working electrodes (Figure 2A) coupled with a 3D-printed flow cell for detecting heavy metal ions, As(III), Cd(II), and Pb(II). The working electrodes were modified with (BiO)_2_CO_3_-rGO-Nafion for Pb(II) and Cd(II) sensing, and Fe_3_O_4_-Au-IL for As(III) detection. The linear range of analytical curves was from 0.0 to 50 μg L^–1^ for all three analytes, with limits of detection (LOD) estimated at 2.4, 1.2, and 0.8 μg L^–1^ for As(III), Pb(II), and Cd(II), respectively. Finally, simulated river water provided 95–101% recovery for the analyzed metal ions, indicating high selectivity and accuracy and promising tools for environmental applications.

Oliveira et al.^93^ proposed a novel carbon-based ink combining graphite flakes and polyester resin, which was possible to create an electrochemical paper-based analytical device (ePAD) (Figure 2B). The feasibility of the ePAD was demonstrated for paracetamol analysis in medicine samples, and blood plasma focused on a pharmacokinetic study. The electroanalytical method exhibited a linear behavior for the concentration range between 1.0 and 60 μmol L^–1^, with an LOD of 0.2 μmol L^–1^ and satisfactory reproducibility (7.7%). The achieved paracetamol concentrations matched labeled values and did not differ statistically from the HPLC results (at a 95% confidence interval). In pharmacokinetic studies, the ePAD measured a maximum paracetamol concentration of 23.58 ± 0.01 μmol L^–1^, with a maximum time of 30 min and a half-life of 2.15 h. The reported study presented noticeable achievements for pharmaceutical applications. The study described by Khosravi et al.^17^ reported a sustainable electrochemical sensor based on textile substrates with Prussian blue and carbon-based conductive ink for detecting glucose in sweat samples (Figure 2C). The working electrode was modified with carbon nanotubes, chitosan, bovine serum albumin, glucose oxidase, and Nafion to allow a sensitive and selective determination of the analyte. Under optimized conditions, the linear range for glucose concentrations was obtained up to 1000 μmol L^–1^, with a sensitivity of 18.41 μA mmol^–1^ cm^–2^. The obtained results presented high stability over 30 days of storage, demonstrating the suitability of the textile-based sensors for applications in precision medicine of glucose-discrepancies diseases.

Recently, Orzari et al.^94^ described a new conductive ink based on carbon black and poly(vinyl alcohol) for detecting epinephrine and α-synuclein, important biomarkers for Parkinson’s disease (Figure 2D). To enhance the device performance, the working electrode was modified with Pd nanoparticles. For epinephrine sensing, the linear behavior ranged from 0.75 to 100 μmol L^–1^ and the LOD was estimated as 0.051 μmol L^–1^. Regarding α-synuclein, the electrode was modified with biological recognition elements to detect α-synuclein through electrochemical impedance spectroscopy. A linear behavior was obtained in the range from 1.5 to 15 μg mL^–1^ in phosphate buffer and from 6.0 to 100 μg mL^–1^ of α-synuclein in blood serum samples, with LODs calculated as 0.13 and 1.3 μg mL^–1^, respectively. Given the versatility of the proposed (bio)sensor, together with the analytical performance in the range applicable to real diagnosis, it is believed that this platform is highly promising for application in complex matrices.

As stated, screen-printing has become an effective method for producing electrochemical sensors. The process benefits of electrode fabrication include using low-cost materials, such as conductive pastes and inks and various substrates, making electrode fabrication suitable for large-scale production. Its versatility enables the creation of multilayered sensor designs that can be customized for specific applications. Additionally, the method can be applied to both flexible and rigid substrates, broadening its potential uses. It provides scalability that is suitable for enhancing more practical benefits, smoothing the transition from prototyping to large-scale production. The technique also accepts the use of automated systems with great efficiency, slightly increasing the amount of resources expended. This is especially advantageous for medical applications that require a more urgent production.

Intricate patterns with robust and durable sensors can be achieved, and the use of chemically resistant materials enhances the robustness of the final devices for harsh analytical conditions. Also, many of the inks and pastes can be developed using eco-friendly materials. Together with the minimal waste production, the screen-printing technique is quite an environmentally safe approach. All things considered, screen-printing is a leading choice for producing high-performance electrochemical sensors due to its mix of functional, financial, and environmental advantages.

Also, with the curing of the ink, the electrode surface is highly susceptible for modifications.^11,95^ Screen-printed devices are commonly modified with metallic and organic (nano)particles, by drop-casting^89,96^ or even electrodeposition.^97,98^ By adding Au-based structures, as an example, the device surface presents biocompatibility spots for the incorporation of many biomolecules. This is an extensively employed approach for the fabrication of immunosensors.^99−102^

Ongoing research in screen-printed electrochemical sensors focuses on enhancing resolution, material compatibility, and performance.^8,32^ Key innovations include using advanced materials, improving resolution through nanoinks and hybrid printing techniques, and developing flexible substrates for wearable applications.^8,32,103^ Researchers are also integrating sensors with digital platforms for real-time monitoring^104^ and enhancing sensitivity with molecularly imprinted polymers^105^ and biorecognition elements.^106^

Despite the advantages mentioned, screen-printing still faces several challenges. While the produced electrodes have a customizable shape, the lack of resolution of the technique can damage detailed geometries and cannot compete with fine designs made by other 2D-based production techniques, such as photolithography, for instance. This can restrict the miniaturization and precision of sensors, regarding performance and sensitivity, when such a detailed architecture is necessary. Additionally, the technique reproduction is dependent on the layer thickness, which can lead to inconsistencies in conductivity, impacting reliability and accuracy.

The technique also has limitations with respect to the compatibility of ink or paste and the substrate. Ideally, the interaction strength between the conductive material and the substrate must be stronger than with itself, with adhesive and cohesive forces playing an important role. It is also important to highlight the flexibility and irregularity of the surface, as the first must be compatible with the composite or will stretch it, leading to structural fissures, while the latter can alter the interaction moment of the ink, facilitating its adhesion. However, this interaction is dependent on the viscosity of the ink. Further, in the matter of ink layer thickness, very viscous composites tend to be cured into thicker layers, which can diminish response time and sensitivity, requiring the addition of more conductive components to achieve usable devices.

When manually producing a batch of electrodes, one must consider the maintenance of the equipment, as many polymeric materials or solvents can provoke chemical attacks on squeegees, spatulas, or other laboratory utensils, resulting in additional expenses. In this mode, a high precision of the handler is also needed for quality control. These points can be averted by the use of mechanical printing. Environmental concerns also arise from the inks and substrates used, although minimizing waste is a known benefit. Lastly, while screen-printing supports rapid prototyping, interactive design changes can be cumbersome due to the need for new screens and setups. Innovations in materials, automation, and flexible printing technologies could address these limitations, expanding the capabilities and applications of screen-printing in sensor production.

In summary, while screen-printed sensors offer several advantages, including cost efficiency, rapid production, and versatility in terms of material compatibility, they also present challenges in terms of resolution, two-dimensionality, batch reproducibility, and substrate integration. Understanding these factors is critical to choosing this technology and optimizing the design and fabrication of SPE devices for specific desired applications.

## Additive Manufactured Electrochemical Sensors

**Figure 3:**
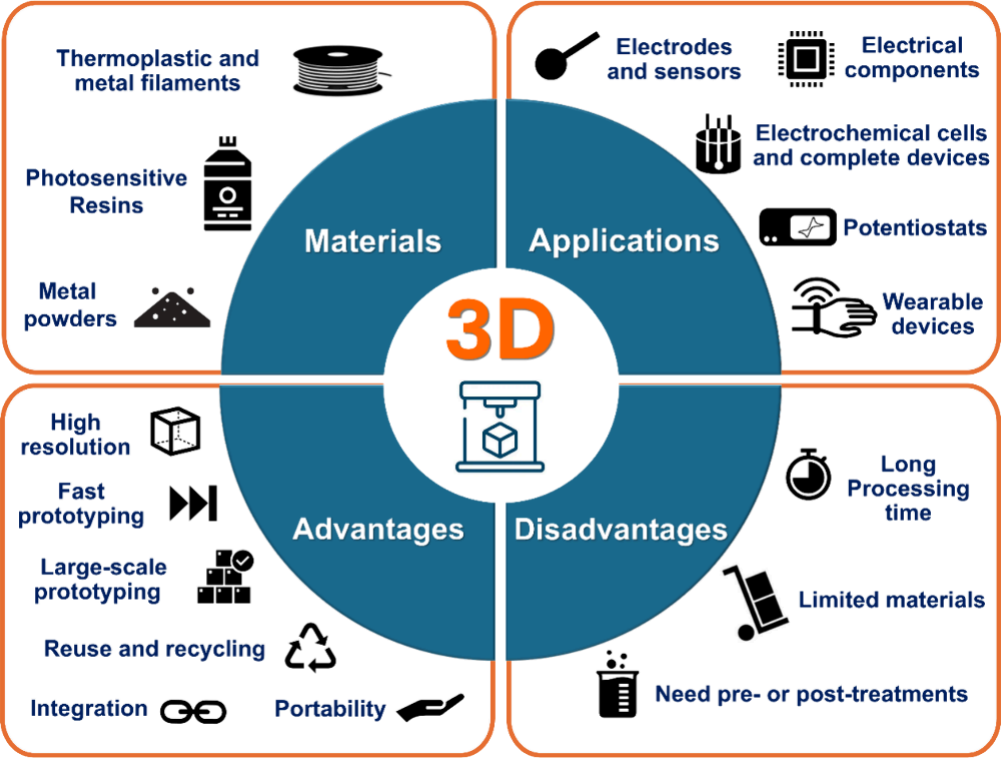
Summary of 3D printing materials, applications, and main advantages and disadvantages.

Collaborative robotics and the 4.0 industrial revolution have brought certain technical advances to electroanalytical instruments such as automated sampling, chemical analysis, and data transfer. Another advantage of such an advancement is device prototyping and manufacture. When considering the most crucial characteristics in the sensor construction process, these must be highlighted: the versatility of a given design, its fabrication time, repeatability, and automation. Among the diverse landscape of fabrication processes, one option may be regarded as a protagonist in the matter: the additive manufacturing technique, commonly referred to as “3D printing”.^9,107−109^Figure 3 summarizes the most commonly employed materials and their applications, advantages, and disadvantages, which will be discussed in detail in this section.

Normally, three important steps are necessary to fabricate 3D printed devices, which are modeling, slicing, and printing processes. The modeling method is the step when the desired project of electrodes is created by using computer-aided design (CAD) software, and the desired project is converted to an STL file. The following step is slicing, in which printing parameters can be selected, such as speed, direction, layer thickness, infill style, and percentage. In some cases, the sliced project needs to be converted to a compatible file, according to the software used. The last step is the printing process, which is when the desired project makes a solid form.^108^ Before the above-mentioned step is started, the printer tool needs to be calibrated, aiming to ensure suitable quality for the printed electrode. More details about the printing process are provided below.

**Figure 4:**
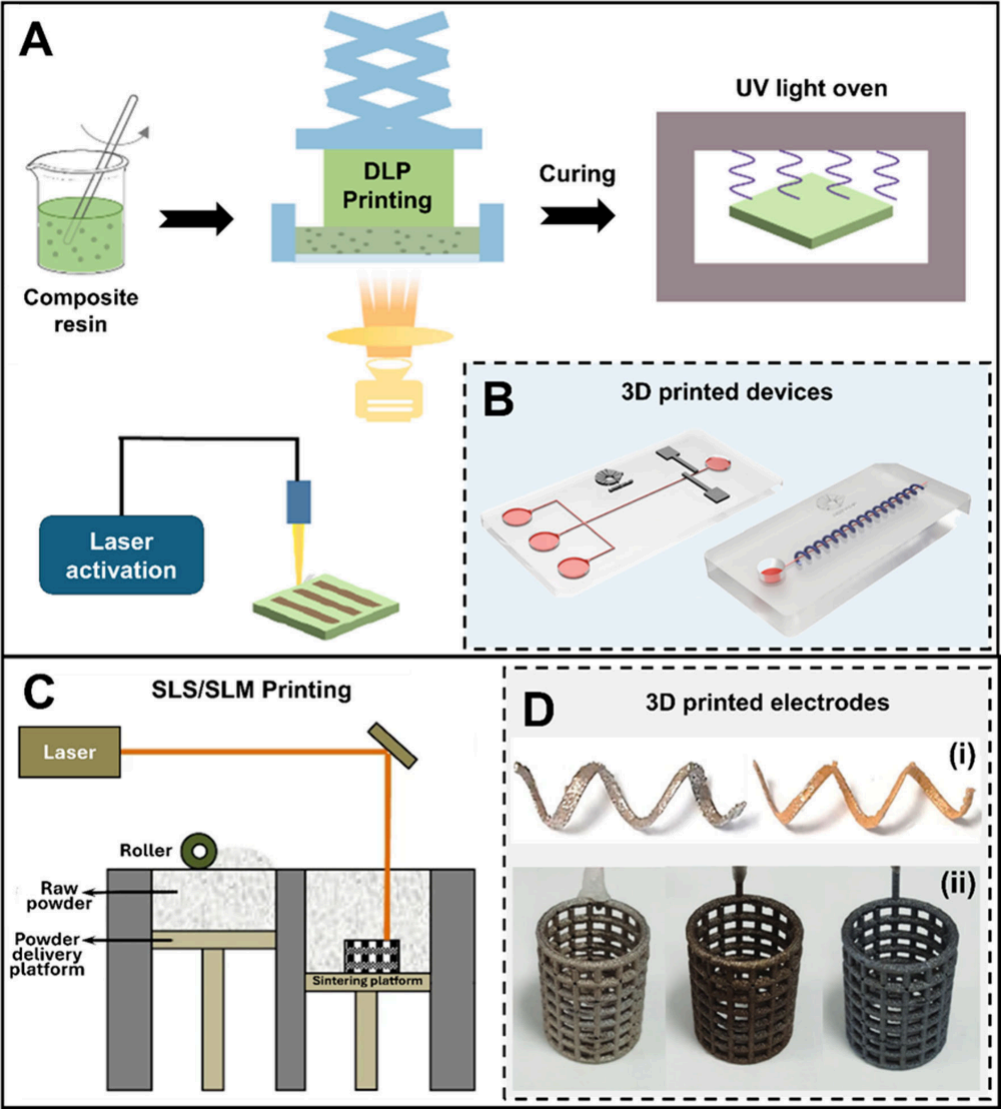
(A) Digital light processing (DLP) using a composite resin, followed by laser activation. (B) 3D printed microfluidic/microchip devices. (C) Selective laser sintering/melting (SLS/SLM) process. (D) 3D printed stainless steel electrodes by SLM before and after gold-plating (i), IrO_2_ and platinum (ii), modifications. Adapted with permission from (A) ref (113), Copyright 2022, MDPI, (B) ref (122), Copyright 2023, RSC, (C) ref (117), Copyright 2020, Springer, (D-i) ref (120), Copyright (2017), ACS, and (D-ii) ref (123), Copyright 2018, Wiley-VCH.

From the point of view of fabrication, different materials and methods can be used, depending on the manufacturing type, which includes photosensitive resins, powders, and thermoplastic filaments. The first method combines liquid photosensitive resins and a light source polymerization process to make a highly defined solid object, which is more explored aiming to create electrochemical cells and prototypes. In this case, different light sources (i.e., ultraviolet, laser, and LED, among others) are employed to cure and photopolymerize the resin.^110^ Stereolithography (SLA) and digital light processing (DLP) are the most used techniques for this purpose, aiming for the production of high-resolution and miniaturized prototypes, such as microfluidic devices.^111^ Transparent, stretchable, and conductive hydrogel-based sensors have been reported, demonstrating the versatility of this technology to develop wearable devices, adaptable to different parts of the human body.^112^ In another work, an electrochemical biosensor for the detection of glucose was fabricated by DLP using a resin modified with copper hydroxide phosphate for the base construction, and the electrodes were obtained by laser activation and coating with copper and silver (Figure 4A).^113^ As seen, polymeric resins and hydrogels can be modified using a variety of materials (e.g., conductive polymers, metals, nanoparticles, fluorescent materials, and others) to improve their conductive properties for the development of electrochemical sensors and cells, lab-on-a-chip, wearables, microfluidic devices, and other systems,^114,115^ as shown in the example of Figure 4B.

Similarly, powder-based additive manufacturing has also been successfully employed for this purpose, combining metal or thermoplastic particles, and a source of high energy (e.g., laser, electron beam, and plasm).^9^ In this approach, the particles are melted and deposited, creating well-defined materials.^110,116^ Although this printing method is scarcely explored to manufacture electrochemical devices, selective laser sintering (SLS) and selective laser melting (SLM) techniques are highlighted for the development of metallic electrodes (e.g., steel, aluminum, nickel, titanium, and copper, among others). For this, a laser is used to merge fine powder particles, which are layer-by-layer deposited to form a 3D solid object by sintering or melting (Figure 4C).^117,118^ Complex-shape electrochemical sensors prototyped by SLM have been described using mostly stainless steel, followed by their easy surface modification by plating/coating methods, using metals and electrocatalysts (e.g., gold, bismuth, platinum, and IrO_2_), as shown in Figure 4D.^116,119−121^ This provides the enhancement of sensor conductivity and electrochemical performance for the detection of pharmaceutical, biological, explosive, and pesticide targets. Thus, this approach allows the construction of high-conductivity metal electrodes with complex and customized designs that can be easily functionalized or modified to meet different electrochemical applications.

The filament-based method has been successfully used for manufacturing electrochemical devices, employing a combination of thermoplastic composites and portable or desktop printers. The fused filament fabrication (FFF) is probably the most employed method due to its advantageous characteristics, such as the easy operation and lower cost of 3D-printers and thermoplastic filaments, compared to other additive manufacturing techniques.^9,124^ It is an extrusion-based technique that can produce printed solid objects using an additive printer, which has as its operating principle the melting of a desired thermoplastic material, followed by layer-by-layer deposition on an inert solid surface. In fact, this type of printer makes use of two interconnected segments, one being a printing nozzle that is loaded with the desired material. The nozzle tool is a metallic compartment where the thermoplastic filament is melted by employing adequate temperatures according to the material (between 180 and 240 °C), which is drained to a specific diameter that can range from 0.1 to 0.6 mm. The most usual option of a nozzle for creating electrodes is 0.2 or 0.4 mm. Values less than 0.1 mm can be more susceptible to obstruction on the nozzle, affecting the printing quality.^125^ Another segment is the printing bed that is used for depositing polymeric layers that can work at room temperature or even hot temperature with values ranging from 40 to 110 °C, depending on the employed filament.^126^

Conductive or nonconductive thermoplastic polymers are used, which mostly include polylactic acid (PLA), acrylonitrile butadiene styrene (ABS), PET, and thermoplastic polyurethane (TPU), among others.^9,127^ These filaments are employed for constructing insulating regions in electrochemical cells. Carbon (i.e., carbon black, carbon nanotubes, graphite, and graphene, among others)- and metal-based materials (i.e., stainless steel, copper, iron, and nickel) have been found as fillers in conductive filaments.^32,123,128−134^ Conductive particles normally have a diameter size ≤400 nm, which is crucial to prevent the filament from blocking the printer nozzle.^32,123,128−130^ A topic discussing different approaches for using additive manufacturing for electrode fabrication, with real examples and a discussion on resource cost, can be found in the Supporting Information.

Along these lines, 3D printing technology has been an allied of electrochemistry research, allowing not only the construction of sensors and biosensors but also electrical components, potentiostats, and miniaturized, integrated, complete devices and systems. Therefore, 3D-printed electrochemical devices provide sensitive and selective approaches toward several targets in biological, pharmaceutical, forensic, food, and environmental applications.^32^ Considering the above-mentioned factors, some advantages and disadvantages of the additive manufacturing technique are highlighted below.

Additive manufacturing is a versatile technique in which the main characteristics are the prototyping customization of shapes and sizes, easy and rapid manufacturing, and energy efficiency, making this an advantageous method for academic and industrial approaches.^9,135,136^ In addition, these processes show advantages over subtractive processes since objects are layer-by-layer printed, using just the required amount of material.^137^ Thus, additive manufacturing has ended up becoming more popular and sustainable since it saves feedstock and generates less waste.

Both commercial and lab-made 3D printing materials (i.e., filaments, resins, and powders, among others) can be easily surface modified by different strategies, which include mechanical, chemical, and electrochemical activations.^128^ In addition, the electrode surface properties can be improved by anchoring nanomaterials, redox mediators, catalyzers, and bioreceptors.^123,138−140^ Among the most common approaches of surface modification for these devices, the literature presents a myriad of reports employing drop casting, sputtering, and electrodeposition.^141−146^ On the other hand, internal modifications have been performed by adding conductive materials (i.e., carbon, metals, and nanomaterials, among others), plasticizers, and other fillers in 3D printing materials, especially in thermoplastic filaments.^22,147,148^ Thermal mixing followed by the re-extrusion process is probably the better way to obtain homogeneous modified filaments.^149^ Although there are commercially available materials for 3D printing, modified feedstock has been required as a way to obtain more efficient materials and devices, allowing the modulation of the materials according to the desired applications. This is advantageous for the development of electrochemical sensors, which improve their electrochemical, physical, and mechanical properties.

The advance of this technology has created the possibility of quickly printing complex, high-resolution prototypes. Binder jetting (or powder bed fusion), selective laser sintering or melting (SLS or SLM), and stereolithography (SLA) are examples of high-accuracy and resolution techniques, providing high-quality printing.^135^ In addition, binder jetting and laser sintering are also highlighted as rapid prototyping techniques, in which the benefit is the possibility of large-scale prototyping, shortening product time and boosting productivity, especially for commercial demand.^150^ These techniques allow the fabrication of electrical contacts, circuits, small parts, electrodes, miniaturized and wearable devices, aiming for the development of point-of-care systems.^151−157^ In addition, the development of all-in-one and lab-on-a-chip approaches deserves to be highlighted, which expands the perspectives about the miniaturization, portability, and accessibility of 3D printed sensing devices in remote areas.^158,159^ These apparatuses can be easily combined to Internet of Things approaches, allowing real-time monitoring and transmitting via wireless or Bluetooth, for example.^160^ Parallel to this, the fabrication of 3D-printed potentiostat equipment is another possibility since the whole integrated electrochemical system can be constructed and adapted as required.^26,161^

From a sustainable point of view, the reuse and recycling of used printed parts and devices and material waste can be considered. This approach has also been encouraged in both academic research and industrial fields, which can be seen as synonymous with the circular economy, with economic and environmentally friendly benefits. This is mostly reported using recycled thermoplastics and metal powders, waste, and old printed parts, among others.^125,162,163^ Parallel to this, the printed devices and sensors can also be recycled and remanufactured to obtain new electrochemical cells, increasing the material life cycle.^149^ Considering the recycling process, the balance between sustainability and print quality should be considered, respecting the limit of remanufacturing cycles without losing the physical and mechanical properties of the final printed parts. Typically, material recycling has been reported for 3–10 cycles for FFF and metal powder in laser powder bed fusion without failures.^164,165^ This demonstrates the facility to fabricate 3D-printed sustainable electrochemical sensors and devices.

In the reign of additive manufacturing electrodes, if one wants to reach popularization and commercialization, there are some important key points to overcome.^166^ These aspects can be classified into three different frontiers: the development of the electrodes, sensing application, and environmental impact. Considering the electrode fabrication, the first question to mention is associated with the cost of printers, modeling software, and the training time of operators. To overcome these barriers, some strategies have been introduced, including sales of disassembled printers, free modeling tools that have limited commands, and collaboration between chemists and engineers, which could result in less time to develop projects and print the devices. Furthermore, the high cost of printer modeling software can negatively impact the popularization of technology, especially in academic research. However, the increase in dissemination and sharing of device designs (STL or other compatible files) should be important to new users to transform good designs into great ones.

Other points to highlight are associated with the availability of conductive materials, especially filaments, and their electrical resistance. Minimal options are commercially available, with PLA-carbon black conductive filament from Proto-Pasta being the most popular for creating carbon-based electrodes. That option of filament exhibited values of electrical resistance ranging from 2000 to 3500 Ω for 10 cm of filament (ϕ = 1.75 mm). Another option is the TPU (thermoplastic polyurethane) conductive Filaflex from Recreus, which presents an electrical resistance value ∼5000 Ω, for 10 cm of filament (ϕ = 1.75 mm). However, the field of FFF is growing with many research groups making lab-made filaments. The electrical conductivity of filaments has been solved by increasing the percentage of conductive fillers in the lab-made filaments, with values ranging from 20 to 30 wt %, resulting in materials with electrical resistance ∼800 Ω, for 10 cm of filament (ϕ = 1.75 mm), without reducing the mechanical resistance, which is also important to keep on active.^22^

The catalytic ability of manufactured electrodes is another point to mention, as it can directly impact the sensing performance of the desired electroanalytical method. This aspect has been achieved by performing pretreatments and/or surface modifications of the electrodes in addition to the fabrication of lab-made filaments with improved electrical conductivity, as discussed previously. In this sense, the mechanical polishing method upon the electrode surface can be an alternative if the users have no access to chemical, electrochemical, or laser protocols.^167,168^ It is important to mention that the above-mentioned activation option can remove roughness from printed devices. Another strategy that can be utilized is to fabricate electrodes with a short connection length.^169^ The anchoring of conductive nanomaterials on electrode surfaces such as gold nanoparticles and graphene, for example, is also reported to improve the performance of sensors.^136^ Another pertinent approach for surface modification is the use of conductive inks, such as the ones mentioned in the screen-printing section, to achieve more interesting performance.^170,171^ Reports such as the ones from Hernández-Rodríguez et al.^171^ have highlighted the employment of a print-pause-print approach, where the final device can be modified between layers. This ensures a more specialized application of the sensor, which is especially useful for FFF-based microfluidics devices.

The environmental impact is a critical point to overcome to stay in the race toward commercialization of 3D-printed electrochemical devices. In this aspect, 3D printing methods using filament melting and liquid resins, for example, can produce some residues such as the emission of ultraparticles and volatile organic compounds during the printing processes at high temperatures. Also, the production of plastic residues is another problem, considering the use of thermoplastic materials. In this way, it is important to propose strategies for reducing or remediating these potentially hazardous materials. Recent studies have successfully reported some strategies that are converging toward sustainability, including the recycling of thermoplastic residues for creating new filaments and electrodes, the adjustment of printing parameters, and the miniaturization of systems, resulting in the reduction of plastic waste for the manufacturing of electrochemical devices.^68,125,172^

## Conclusion and Outlook

This revision aimed to compare both screen-printing and additive manufacturing techniques, showing their advantages and challenges when using them to produce electrochemical sensors. Being a strong contender for large-scale fabrication of such devices, screen-printing is an already established process, with incomparable cost-effectiveness, that can act in approaches that desire fast, simple, but reliable electrodes. This is a technique that can produce devices with reproducible geometry and is more appropriate for low-volume sensors. On the other hand, additive manufacturing is a very precise method of fabrication that enables very complex geometries to be produced while maintaining interesting mechanical properties, such as flexibility and wettability. When customized sensors are required, additive manufacturing, with its many different approaches and materials, can be the go-to protocol for easy prototyping. With the integration of multiple printing technologies and heterogeneous materials, innovative devices can be produced with great robustness and quickly updated, if desired.

When the most appropriate techniques for the fabrication of a given electrode system are selected, the following questions should be asked: What are the better viscosities and sizes of carbon-based flakes for creating conductive ink? What level of print resolution is needed for additive manufacturing of electrodes? What are the specific applications of this device? Is a very high sensitivity really necessary? What is the desired determination range? Is the sample or other analysis environment chemically aggressive? How much short-term and long-term resources can be spent to produce this electrochemical sensor? To increase sensitivity, the researcher may need to add different particles, change electrode geometry, employ modern machines, use different chemical or electrochemical treatments, and increase the time and resources cost. Still, it is important to highlight that in some cases, a less sensitive device could have perfectly achieved the desired goals, for both printing processes. In a particular topic of resources, both techniques have very different demands. Screen-printing can be performed even without high technological equipment, and with little training anyone can produce very reproducible electrodes. The immediate cost needed to achieve impactful results with additive manufacturing is considerably higher. Resources must be spent on training personnel in different software (including modeling, slicing, and printing computational programs) and in printing, extruding, or other machines. However, no other electrode fabrication technique allows for the freedom of design that this method does. Very specific current healthcare challenges could be solved by tailor-made additive-manufactured electrode systems, integrating biocompatibility with the flexibility of a conductive filament, for instance.

**Figure 5:**
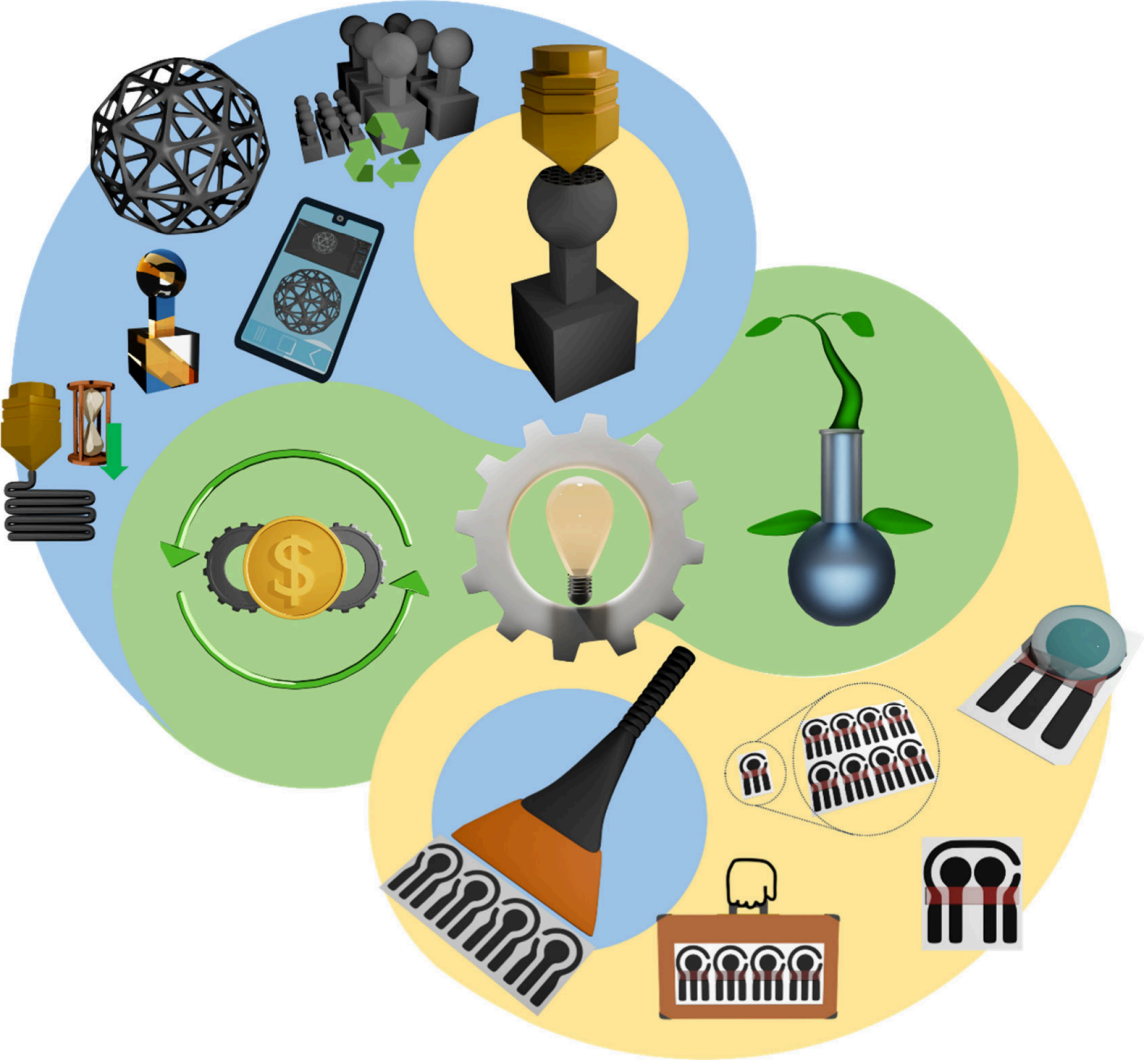
Graphical representation of the advantages of screen-printing and additive manufacturing for the fabrication of electrochemical devices. Advantages for additive manufacturing (blue segment), from left to right, top to bottom: rapid prototyping, complex three-dimensional geometries, multiple material for printing, reuse, or recycling, technological integration, scalability, and mass production. Advantages for screen-printing (yellow segment), from left to right, top to bottom: portability, scalability, and mass production, multiplex devices, and low sample volume for analysis. For both techniques (green segment), from left to right: high cost–benefit, high customization and biocompatibility.

As a short summary, Figure 5 is a graphical representation of the more impactful qualities of both techniques. Additive manufacturing has the following concepts as its main attractions: it is known for rapid prototyping, multiple and different materials can be used to print complex geometries, it can be tailor-made to solve specific problems, and it has a high integration with modern-day technologies. While sometimes slower than screen-printing, the materials can also be mass-produced, and the most commonly employed filaments can be reused or even recycled. The strengths of screen-printing, on the other hand, can be highlighted as portability, ease of producing multiple electrodes in a very short time, with small sample volume usage, and the possibility of multiple analyte determination with multiplex devices. The two have high cost-effectiveness and customizability. Depending on the materials used, both present interesting biocompatibility capabilities.

The past few years have demonstrated that both technologies, screen- and additive printing, still have challenges to overcome, and this review discusses these key points, side-by-side. When one looks for the future, it is possible that research focused on mitigating the impact of such challenges may appear. Even though screen-printing can mass-produce quite reliable devices, there is still a lack of versatility in such sensors in terms of novel materials and their functionalities. The integration of printed sensors into other equipment is also a truculent step in using these sensors. For additive manufacturing, there is a concentration on using FFF-based devices, especially due to cost, but there is much to be discovered about the practicality of other printing methods in this field. Also, the most important aspect of additive manufacturing is scarcely researched in the literature: the geometry of the electrodes. The authors here incentivize any reader to explore the potentiality of three-dimensional working electrodes, using mathematically controlled high-surface contact geometries to increase sensitivity. Additionally, both techniques discussed in this paper can be integrated. We sincerely hope that highly efficient devices can be produced by integrating screen-printing and additive manufacturing, highlighting the qualities of the two worlds and driving forward the capabilities and potential applications of various fields.

## Supporting Information Available

Additional topical discussion (PDF)
