# Supplementary material for: Screen-Printing vs Additive Manufacturing Approaches: Recent Aspects and Trends Involving the Fabrication of Electrochemical Sensors
Source: Anal Chem. 2025 Jan 16;97(3):1482–94. doi: 10.1021/acs.analchem.4c05786 (PMC11780578; doi:10.1021/acs.analchem.4c05786)
Supplement: Supplementary file 1 — ac4c05786_si_001.pdf [file ac4c05786_si_001.pdf]

Supporting Information

Screen-printing vs. additive manufacturing approaches: recent aspects and trends involving the fabrication of electrochemical sensors

Luiz O. Orzari[a,b]; Cristiane Kalinke[c,d]; Habdias A. Silva-Neto[f]; Danielly S. Rocha[e]; Jéssica R. Camargo[a,b]; Wendell K.T. Coltro[e,g]; Bruno C. Janegitz[a,*]

[a] *Department of Nature Sciences, Mathematics and Education, Federal University of São Carlos, 13600-970, Araras, SP, Brazil*

[b] *Department of Physics, Chemistry and Mathematics, Federal University of São Carlos, 18052-780, Sorocaba, SP, Brazil*

[c] *Institute of Chemistry, University of Campinas, 13083-859, Campinas, SP, Brazil*

[d] *Department of Chemistry, Federal University of Parana, 81531-980, Curitiba, PR, Brazil*

[e] *Institute of Chemistry, Federal University of Goiás, 74690-900, Goiânia, GO, Brazil*

[f] *Department of Chemistry, Federal University of Santa Catarina, 88040-900, Florianópolis, SC, Brazil*

[g] *National Institute of Bioanalytical Science and Technology, 13084-971, Campinas, SP, Brazil*

*Corresponding author: brunocj@ufscar.br

THE IMPORTANCE OF SCREEN-PRINTING AND ADDITIVE MANUFACTURING IN THE ACADEMY, THE ENVIRONMENT AND THE INDUSTRY

Many printing techniques have a wide range of applications for humankind, and its no different for screen-printing and additive manufacturing. In the academia, screen-printing commonly used to produce electronics circuits [1,2], sensors [3,4] and other diagnostics devices [5,6]. It allows for quick prototyping and experimentation, and, by employing low-cost materials, it is a valuable tool for engineering and biotechnology [7,8]. Additive manufacturing, is even more used for prototyping, as it enables the production of very elaborated and quick-to-create models for testing. It is incentivized in the customization of prosthetics and implants in medical research [9,10], as well as educational models, helping to visualize advanced concepts, especially is the disciplines of natural science [11,12].

These printing techniques also have contributed to the environmental sustainability. Screen-printing is often used in solar cells [13-15], water filtration systems [16] and eco-friendly packaging [17,18], providing a great cost-benefit. Additive manufacturing can help in the reduction of waste by using a calculated amount of material in production, while also being known for many reusing efforts, creating new products from used components [19,20]. Filaments can also be made from recycled materials, and this is encouraged by sustainable construction practices, such as 3D printing homes with recycled materials [21,22].

With mass-production capabilities, screen-printing has gained a large space in industrial sectors, specifically on the production of textiles [23,24], packaging [25,26] and electronic circuits [27-29], much like in the academy. It is also commonly employed in the production of advertising objects, such as cards, labels, tags and the like. However, the greater achievement of additive manufacturing in the industry cannot be overlooked, as it is considered by some as a novel industrial revolution [30,31]. It allows for the creation of parts, tools, and many products on demand. This reduces the large inventories, while minimizing production time, with confirmed improvements on industries like healthcare [32], automotive [33] and even aerospace [34].

Both printing techniques have, in recent years, provided innovation, cost-effectiveness and optimization of different protocols across the globe, leading the technological advancement of our society.

COMPARATIVE ANALYSIS OF SCREEN PRINTING AND OTHER TWO-DIMENSIONAL SENSOR FABRICATION TECHNIQUES

Cost-effectiveness and scalability are very attractive qualities of screen-printing, making it very suitable for the production of flexible sensors with a large active area [35]. Its affordability is a determinant factor when considering investing in a research project, especially on lower budget and/or aiming relative low-cost systems [35,36]. Parties that target environmental monitoring [37,38], wearable electronics [39,40] and consumer-friendly devices [41,42], have a high interest in this method of fabrication. The extensive use of substrates also cannot be ignored, as they add versatility to the final device. The overall process is quite simple, allowing for small business, startups and the like to also have interest in this printing technique [35].

It is important, however, to understand the current limitations of the technique. Screen-printing struggles with high-resolution and precision, with many research groups focusing on improving these very same properties of the process [43]. Conductive inks can also be less chemically resistant, with organic samples being potentially destructive for the device [44,45]. Sensitivity can also be of concern, as in carbon-based inks, for instance, the layer thickness can be responsible for increasing electrochemical resistance [44,45]. Additionally, screen-printing favors simple designs, with multi-layered structures or complex nano-scale geometries requiring additional effort on researchers, being it the investment of more precise machinery or even the invention of novel apparatus to support the desired system [44,45].

However, when compared to other techniques, screen-printed devices present distinct trade-offs. For instance, photolithography and laser direct structuring provides extremely high resolution [46-48] and are able to produce very complex sensor designs, making them ideal for medical diagnostics. Although, both techniques require specialized personnel, equipment and practically demands a cleanroom facility. Photolithography is also very time- and resource-consuming. While the later technique is very scalable, it is, together with the former, less accessible than screen-printing [46-48].

Being a similar method, inkjet printing also provides high resolution and the possibility of flexibility for the final device, but is considerably slower and can be more expensive than screen-printing, depending if and which machinery is used [49].

When the obtention of resources is a challenge, screen-printing is very valuable due to its large-scale and low-cost production, but the method is indeed limited for its resolution and performance, when unmodified. The question on which production technique to choose in an intriguing one, as all the mentioned alternatives offer very attractive properties. The ultimate answer depends on the balance between cost, performance and scalability, and the right option is the one that allows for your goal to be fine-tuned and, in the long run, flourish.

DIFFERENT WAYS TO PRODUCE ELECTROCHEMICAL SYSTEMS WITH ADDITIVE MANUFACTURING

Nowadays, it is possible to manufacture additive printed electrodes by using three different protocols, including single and multi-material extrusion employing desktop printers and utilizing a portable 3D pen. The first option makes use of printers on single extrusion mode aiming to fabricate separate electrodes using conductive filaments, or electrochemical cells using non-conductive filaments. In some cases, electrodes are also manufactured via additive printing, and the electrochemical cell is manually assembled. Normally, the cost to purchase a desktop printer with a single extrusion is inferior to 2000 USD [50]. It is important to mention that the sector is growing relatively fast, and it is possible to obtain 3D printers for less than 300 USD. However, some key points such as print quality and maintenance cost are issues to overcome. For example, a working electrode was manufactured using an additive printer in a single extrusion mode and PLA-carbon black filament (Figure 1A-i) [51]. The key achievement of that study was the fabrication and assembly of a complete rotating disk electrode (RDE) system. ABS filament was also employed, aiming to create insulating regions of the electrode. The RDE resulted in a total cost of ~100 USD and it was successfully applied for the detection of L-DOPA drug.

For the fabrication of full electrochemical cells or all-in-one devices, multi-material extrusion can be the finest option[52-55]. This can be achieved by using a 3D printer composed of multiple nozzles. These printers are capable of interleaving conductive and insulating filaments, creating a complete device, including all electrodes and the desired region to sample addition. That printer equipped with dual extrusion of filaments can be obtained by investing around 3000 USD. Some key points need to be overcome if such printer is desired for electrode fabrication, especially when thermoplastic filaments are utilized with different melting temperatures and electrical resistivities. Thus, the choice of filaments with similar melting temperatures is important to obtain greater performance in terms of printing quality and reproducibility. Regarding electrical resistivity, printers with two separate nozzles can be used. Another option is using one printer equipped with

dual extrusion mode (side-by-side filament) in the same nozzle. In this strategy, the printing process needs to be performed in the presence of a cleaning step that is selected after changing each filament (conductive or non-conductive). This can be done in the slicing software, resulting in an additional process in the automatic device manufacturing protocol. A common electrochemical cell with three electrodes (reference, auxiliary, and working electrodes), electrical contacts, and sample addition recipient is successfully reported (Figure 1A-ii) by Duarte et al.[56]. For creating the device, a multi-material printer using PLA-carbon black and ABS commercial filaments ($\varnothing$ = 1.75 mm) was utilized, resulting in a fabrication time of ~12 min, manufacturing cost of ~0.08 USD, and 200 µL of sample volume, per device.

Parallel to the use of commercial filaments, the fabrication of sensors has leveraged research focused on new bespoke conductive and non-conductive filaments, allowing the fabrication of competitive, more efficient materials for sensors. Thermal mixing and extrusion of PLA recycled filaments have been reported, followed by the FFF printing of a small volume (200 µL) electrochemical cell using recycled PLA, auxiliary and pseudo-reference electrodes coupled to the cell, and a removable working electrode (Figure 1B) [57]. The conductive filament was composed of 65 wt% recycled PLA, 10 wt% plasticizer, and 25 wt% carbon-based materials (carbon black and carboxylated multi-walled carbon nanotubes). The working electrode was surface modified with yellow fever virus genetic material, aiming at the selective detection of the virus in biological samples.

In another work, a lab-made thermoplastic material was successfully manufactured by using the fused filament fabrication method (FFF) aiming to create a compact BIA cell (5.0 mL). To create the conductive filament based on PLA, carbon black, and plasticizer is proposed for both static or hydrodynamic analyses (Figure 1C-iii) [58]. The complete electrochemical cell containing a pipette tip showed low cost of printable materials (~2.7 USD). The working electrode was surface activated to be used as a catalytic material, aiming at the determination of atropine in forensic samples. Thus, the mixture of carbon-based materials with thermoplastics and plasticizer has been widely reported in the literature for the fabrication of highly conductive, flexible, cost-effective, biocompatible, and sustainable filaments [57,59-62]. Furthermore, it is important to highlight that bespoke filaments can be produced and modified using diverse materials and compositions, which directly affects their final properties [63]. For example, excess of conductive materials can affect the mechanical properties of the filaments negatively, while the presence of plasticizers can improve them. Thus, the evaluation of mechanical,

physical, and chemical properties of the filaments is crucial to achieving high-quality electrochemical devices.

The mixing of printing techniques is also of high interest and deserves to be emphasized. As an example, a ring-shaped wearable sensor has been developed by FFF using TPU and carbon-PLA filaments, aiming the monitoring of glucose in sweat [64]. The working electrode was coated with a gold film to improve the electrochemical performance of the biosensor. Also, the device was coupled to a miniaturized potentiostat connected to a smartphone, making it a completely portable system (Figure 1C-iv). In another study, the cell top and bottom covers were printed by stereolithography (SLA)/digital light processing (DLP), while the electrodes were fabricated by FFF using BSA filament. After, the electrodes were coated with conductive graphite (55 wt%) and BSA filament [65]. Silicone cover and base were also used for the sandwich-like assembling of the electrochemical cell (Figure 1C-v). The sensor was applied for the determination of paracetamol in pharmaceutical samples, showing better electrochemical performance compared to a commercial filament-based sensor.

In addition to traditional FFF printing using desktop printers, portable printers, such as 3D pens, have proven to be a cheaper and simpler option. Combining a single filament and a 3D pen, the sensor is constructed via a manual 3D printing process. As informed above, the FFF technique allows the manufacturing of different architectures of electrodes, including some segments of electrochemical cells and fully devices. This approach is revealed to be easier for the fabrication of electrodes and sensors, which can also be combined with the traditional FFF manufacturing, aiming at the development of complete electrochemical cells. For example, a 3D pen in association with commercial PLA-carbon black filament was used for the fabrication of electrodes, which were employed as catalytic materials (Figure 1D)[66]. A lab-made template composed of poly(methyl methacrylate) (PMMA) and an electrical contact were developed to support the sensor. The main positive gain of the reported device was the use of a portable printer, allowing the developed systems to perform attractive analyses of environmental and food samples with high cost-benefit.

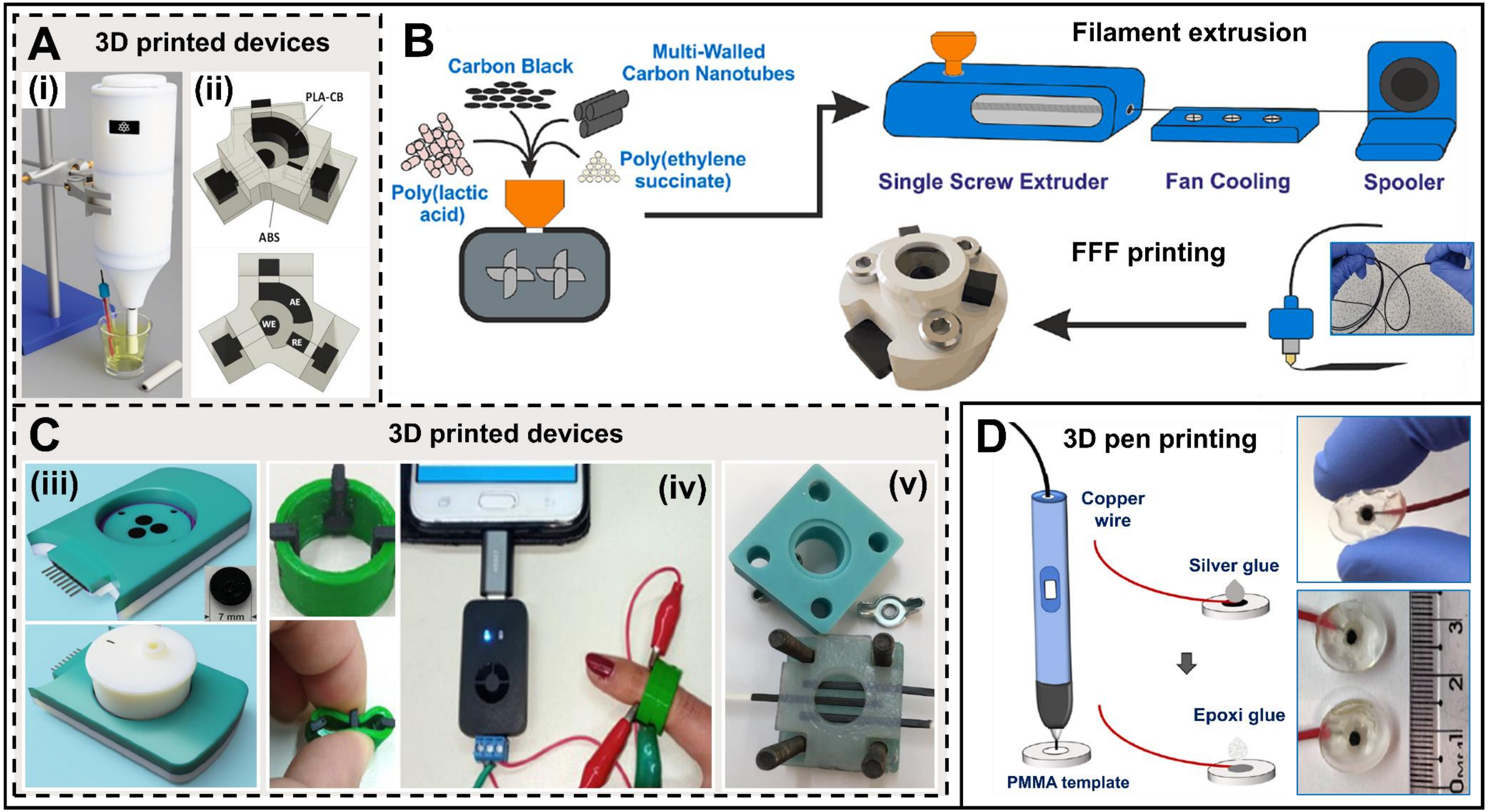
A
3D printed devices
(i)
(ii)
PLA-CB
ABS
AE
WE
RE
B
Carbon Black
Multi-Walled
Carbon Nanotubes
Poly(lactic
acid)
Poly(ethylene
succinate)
Filament extrusion
Single Screw Extruder
Fan Cooling
Spooler
FFF printing
C
3D printed devices
(iii)
7 mm
(iv)
(v)
D
3D pen printing
Copper
wire
Silver glue
Epoxi glue
PMMA template

Figure 1. (A) 3D printed devices: Rotating disk electrode (RDE) system for the detection of L-DOPA (i); and fully electrochemical cell for sequential analysis of hormones (ii). (B) Extrusion for the fabrication of flexible filaments and FFF printing of an electrochemical device for the detection of yellow fever virus. (C) 3D printed devices: Compact BIA cell with a pipette tip for the detection of atropine in forensic samples (i); ring-shape wearable device for the detection of glucose in sweat (iv); and mixed material and printing techniques device for the detection of paracetamol in pharmaceutical samples (v). (C) 3D pen printing process for the manufacturing of a sensor for the detection of environmental and food analytes. Adapted with permission from M. J. Whittingham et al. Analytical Chemistry, 94 (39), 13540-13548, Copyright (2022), ACS 21(A-i) [51]; L. C. Duarte et al. Sensors and Actuators B: Chemical, 364, 131850, Copyright (2022), Elsevier 23 (A-ii) [56]; C. Kalinke et al., Chemical Engineering Journal, 467, 143513, Copyright (2023), Elsevier 24 [67]; (B); I. V. S. Arantes et al. Applied Engineering Materials, 1, 2397-2406, Copyright (2023), ACS 25 (C-iii) [58]; V. Katseli et al. Analytical Chemistry, 93, 3331-3336, Copyright (2021), ACS 29 (C-iv) [64]; and J. M. Petroni et al. Analytica Chimica Acta, 1167, 338566, Copyright (2021), Elsevier 30 [68]; (C-v); L. A. Pradela-Filho et al. Analytical Chemistry, 95, 10634-10643, Copyright (2023), ACS 31(D) [69]

REFERENCES

(1) Eshkeiti, A.; Reddy, A. S. G.; Emamian, S.; Narakathu, B. B.; Joyce, M.; Joyce, M.; Fleming, P. D.; Bazuin, B. J.; Atashbar, M. Z. *IEEE transactions on components, packaging and manufacturing technology* **2015**, *5*, 415-421.
(2) Zavanelli, N.; Yeo, W.-H. *ACS omega* **2021**, *6*, 9344-9351.
(3) Guo, M.; Chen, Y.; Mo, X.; Sun, K.; Du, Y.; Hu, F. *Microchemical Journal* **2024**, *204*, 111103.
(4) Wawrzyniak, M.; Bourkadi, L. E. L.; Peyrard, M.; Rezig, A.; IEEE, pp 1-4.
(5) Spiehl, D.; Schwall, G.; Post, F.; Weber, C.; Dörsam, E.; Blaeser, A.; Kempf, V. A. J.; Hogardt, M. *Biosensors and Bioelectronics: X* **2024**, *21*, 100557.
(6) Wang, X.; Wu, G.; Zhang, X.; Lv, F.; Yang, Z.; Nan, X.; Zhang, Z.; Xue, C.; Cheng, H.; Gao, L. *Advanced Materials* **2024**, 2410312.
(7) Mor, S.; Solanki, S.; Dhull, V. *International Journal of Intelligent Enterprise* **2024**, *11*, 57-72.
(8) Hong, K. H. *Journal of Natural Fibers* **2024**, *21*, 2424930.
(9) Sun, C.; Tang, L.; Liu, T.; Wang, L.; Tian, X.; Liu, C.; Li, D. *Composites Part B: Engineering* **2024**, *281*, 111518.
(10) Maroti, P.; Schlegl, A. T.; Nagy, B.; Toth, L.; Bogar, P.; Jozsa, G.; Rendeki, S.; Mallakpour, S.; Hussain, C. M. In *Medical Additive Manufacturing*; Elsevier, 2024, pp 179-207.
(11) Kantaros, A.; Petrescu, F. I. T.; Abdoli, H.; Diegel, O.; Chan, S.; Iliescu, M.; Ganetsos, T.; Munteanu, I. S.; Ungureanu, L. M. *Applied Sciences* **2024**, *14*, 2550.

(12) Anđić, B.; Lavicza, Z.; Ulbrich, E.; Cvjetićanin, S.; Petrović, F.; Maričić, M. *Journal of Biological Education* **2024**, *58*, 795-811.
(13) Chang, Y. C.; Zhang, Y.; Wang, L.; Wang, S.; Wang, H.; Huang, C. Y.; Chen, R.; Chan, C.; Hallam, B. *Progress in Photovoltaics: Research and Applications* **2024**.
(14) Worsley, C.; Potts, S.-J.; Hughes, D.; Tsoi, W. C.; Watson, T. *Materials Advances* **2024**, *5*, 4354-4365.
(15) Khan, S.; Noman, M.; Khan, A. D. *Optical and Quantum Electronics* **2024**, *56*, 1424.
(16) Farhadipour, M.; Naghdi, B.; Roghabadi, F. A.; Gorgani, A. S. *Desalination* **2024**, *584*, 117766.
(17) Palmieri, E.; Cancelliere, R.; Maita, F.; Micheli, L.; Maiolo, L. *RSC advances* **2024**, *14*, 18103-18108.
(18) Hakim, L.; Deshmukh, R. K.; Lee, Y. S.; Gaikwad, K. K. *Sustainable Food Technology* **2024**.
(19) Spirio, A.; Arrigo, R.; Frache, A.; Tuccinardi, L.; Tuffi, R. *Journal of Environmental Chemical Engineering* **2024**, *12*, 112474.
(20) Tadi, S. P.; Maddula, S. S.; Mamilla, R. S. *Renewable and Sustainable Energy Reviews* **2024**, *189*, 113961.
(21) Yousaf, A.; Al Rashid, A.; Polat, R.; Koç, M. *Sustainable Materials and Technologies* **2024**, e01103.
(22) Atsani, S. I.; Sing, S. L. *Polymers* **2024**, *16*, 2324.
(23) Yildiz, Z.; Kartal, I.; Kocak, E. D.; Ozer, B.; Kus, B. N.; Eryilmaz, O. *Journal of Cleaner Production* **2024**, *448*, 141635.
(24) Costa, N. G.; Buga, C. S.; Homem, N. C.; Paleo, A. J.; Sencadas, V.; Viana, J. C.; Gonzales, A.; Antunes, J. C.; Rocha, A. M. *Journal of Electroanalytical Chemistry* **2024**, 118805.
(25) Kamalasekaran, K.; Sundramoorthy, A. K. *RSC advances* **2024**, *14*, 27957-27971.
(26) Chelliah, R.; Park, Y.; Jeong, Y.-J.; Vijayalakshmi, S.; Barathikannan, K.; Yeon, S.-J.; Lim, M.-j.; Kim, D.-G.; Oh, D.-H. *가가가가가가가가가가가가* **2024**, *39*, 357-377.
(27) Lall, P.; Kulkarni, S.; Miller, S.; IEEE, pp 1-10.
(28) Kim, J.; Choi, H. W.; Kim, B.; Kim, E.; Kim, J. *The International Journal of Advanced Manufacturing Technology* **2024**, *134*, 127-137.
(29) Lall, P.; Kulkarni, S.; Miller, S.; American Society of Mechanical Engineers, p V001T006A010.
(30) Bennetts, D.; Duer, J.; Kandi, R.; Koluprolu, S.; Fatima, K.; Siddique, W. A.; Idrisi, A. H.; IEEE, pp 1-7.
(31) Jafar, M. R.; Tripathi, N. M.; Yadav, M.; Nasato, D. S. In *Advances in Pre-and Post-Additive Manufacturing Processes*; CRC Press, pp 213-230.
(32) Peron, M.; Saporiti, N.; Shoeibi, M.; Holmström, J.; Salmi, M. *International Journal of Operations & Production Management* **2024**.
(33) Abedsoltan, H. *Polymer Engineering & Science* **2024**, *64*, 929-950.
(34) Srivastava, M.; Jayakumar, V.; Udayan, Y.; Sathishkumar, M.; Muthu, S. M.; Gautam, P.; Nag, A. *Applied Materials Today* **2024**, *41*, 102481.
(35) Suresh, R. R.; Lakshmanakumar, M.; Arockia Jayalatha, J. B. B.; Rajan, K. S.; Sethuraman, S.; Krishnan, U. M.; Rayappan, J. B. B. *Journal of Materials Science* **2021**, *56*, 8951-9006.
(36) Li, M.; Li, Y.-T.; Li, D.-W.; Long, Y.-T. *Analytica chimica acta* **2012**, *734*, 31-44.
(37) Choudhari, U.; Jagtap, S.; Ramgir, N.; Debnath, A. K.; Muthe, K. P. *Reviews in Chemical Engineering* **2023**, *39*, 1227-1268.
(38) Rubino, A.; Queirós, R. *Talanta Open* **2023**, *7*, 100203.

(39) Wang, Y.; Pan, W.; Leong, K. W.; Xu, X.; Dong, G.; Ye, X.; Zhang, M.; Leung, D. Y. C. *Journal of Energy Storage* **2023**, *63*, 106983.
(40) Park, S.; Ban, S.; Zavanelli, N.; Bunn, A. E.; Kwon, S.; Lim, H.-r.; Yeo, W.-H.; Kim, J.-H. *ACS Applied Materials & Interfaces* **2023**, *15*, 2092-2103.
(41) Zhu, D.; Fu, S.; Zhang, X.; Zhao, Q.; Yang, X.; Man, C.; Jiang, Y.; Guo, L.; Zhang, X. *Trends in Food Science & Technology* **2024**, 104485.
(42) Verma, A.; Sarwa, K. K. In *3D Printing and Microfluidics in Dermatology*; CRC Press, 2025, pp 303-327.
(43) Zuo, Z.; Zhang, H.; Gao, S.; Wang, C.; Chen, W.-T.; Hu, G. *Journal of Environmental Chemical Engineering* **2024**, 113263.
(44) Zheng, H.; Guo, Z.; Zhu, W.; Li, D.; Pu, Z. *The International Journal of Advanced Manufacturing Technology* **2023**, *128*, 2813-2824.
(45) Paimard, G.; Ghasali, E.; Baeza, M. *Chemosensors* **2023**, *11*, 113.
(46) Bathaei, M. J.; Singh, R.; Mirzajani, H.; Istif, E.; Akhtar, M. J.; Abbasiasl, T.; Beker, L. *Advanced Materials* **2023**, *35*, 2207081.
(47) Wang, P.; Ma, X.; Lin, Z.; Chen, F.; Chen, Z.; Hu, H.; Xu, H.; Zhang, X.; Shi, Y.; Huang, Q. J. N. C. **2024**, *15*, 887.
(48) Zhang, Y.; Wang, X.; Yan, K.; Zhu, H.; Wang, B.; Zou, B. *Advanced Functional Materials* **2023**, *33*, 2211272.
(49) Cao, T.; Yang, Z.; Zhang, H.; Wang, Y. *Heliyon* **2024**, *10*.
(50) Saggiomo, V. *Advanced Science* **2022**, *9*, 2202610.
(51) Whittingham, M. J.; Crapnell, R. D.; Banks, C. E. *Analytical Chemistry* **2022**, *94*, 13540-13548.
(52) Crapnell, R. D.; Bernalte, E.; Ferrari, A. G.-M.; Whittingham, M. J.; Williams, R. J.; Hurst, N. J.; Banks, C. E. *ACS Measurement Science Au* **2021**.
(53) Quero, R. F.; Costa, B. M. d. C.; da Silva, J. A. F.; de Jesus, D. P. *Sensors and Actuators B: Chemical* **2022**, *365*, 131959.
(54) Raj, A.; Chandrakar, A. S.; Tyagi, B.; Jain, A.; Gupta, H.; Bhardwaj, L.; Goyal, A.; Layal, P. K.; Rajora, A.; Malik, G.; Sahai, A.; Sharma, R. S. *International Journal on Interactive Design and Manufacturing (IJIDeM)* **2024**, *18*, 627-648.
(55) Bin Hamzah, H. H.; Keattch, O.; Covill, D.; Patel, B. A. *Scientific Reports* **2018**, *8*, 9135.
(56) Duarte, L. C.; Baldo, T. A.; Silva-Neto, H. A.; Figueredo, F.; Janegitz, B. C.; Coltro, W. K. T. *Sensors and Actuators B: Chemical* **2022**, *364*, 131850.
(57) Kalinke, C.; Crapnell, R. D.; Sigley, E.; Whittingham, M. J.; de Oliveira, P. R.; Brazaca, L. C.; Janegitz, B. C.; Bonacin, J. A.; Banks, C. E. *Chem. Eng. Journal* **2023**, *467*, 143513.
(58) Arantes, I. V. S.; Crapnell, R. D.; Whittingham, M. J.; Sigley, E.; Paixão, T. R. L. C.; Banks, C. E. *ACS Applied Engineering Materials* **2023**, *1*, 2397-2406.
(59) Kechagias, J.; Chaidas, D. *Materials and Manufacturing Processes* **2023**, *38*, 933-940.
(60) Herrero, M.; Peng, F.; Núñez Carrero, K. C.; Merino, J. C.; Vogt, B. D. *ACS Sustainable Chemistry & Engineering* **2018**, *6*, 12393-12402.
(61) Crapnell, R. D.; Kalinke, C.; Silva, L. R. G.; Stefano, J. S.; Williams, R. J.; Munoz, R. A. A.; Bonacin, J. A.; Janegitz, B. C.; Banks, C. E. *Materials Today* **2023**, *71*, 73-90.
(62) Gullo, L.; Mazzaracchio, V.; Colozza, N.; Duranti, L.; Fiore, L.; Arduini, F. *Electrochimica Acta* **2024**, *482*, 143982.
(63) Sharma, A.; Faber, H.; Khosla, A.; Anthopoulos, T. D. *Materials Science and Engineering: R: Reports* **2023**, *156*, 100754.
(64) Katseli, V.; Economou, A.; Kokkinos, C. *Analytical Chemistry* **2021**, *93*, 3331-3336.

(65) Petroni, J. M.; Neves, M. M.; de Moraes, N. C.; Bezerra da Silva, R. A.; Ferreira, V. S.; Lucca, B. G. *Analytica Chimica Acta* **2021**, *1167*, 338566.
(66) Pradela-Filho, L. A.; Veloso, W. B.; Medeiros, D. N.; Lins, R. S. O.; Ferreira, B.; Bertotti, M.; Paixão, T. R. L. C. *Analytical Chemistry* **2023**, *95*, 10634-10643.
(67) Kalinke, C.; Crapnell, R. D.; Sigley, E.; Whittingham, M. J.; de Oliveira, P. R.; Brazaca, L. C.; Janegitz, B. C.; Bonacin, J. A.; Banks, C. E. *Chemical Engineering Journal* **2023**, *467*, 143513.
(68) Petroni, J. M.; Neves, M. M.; de Moraes, N. C.; da Silva, R. A. B.; Ferreira, V. S.; Lucca, B. G. *Analytica Chimica Acta* **2021**, *1167*, 338566.
(69) Pradela-Filho, L. A.; Veloso, W. B.; Medeiros, D. N.; Lins, R. S. O.; Ferreira, B.; Bertotti, M.; Paixão, T. R. L. C. *Analytical Chemistry* **2023**, *95*, 10634-10643.
